# Development of a nomogram for prognostic prediction of lower‐grade glioma based on alternative splicing signatures

**DOI:** 10.1002/cam4.3530

**Published:** 2020-10-13

**Authors:** Yaning Wang, Zihao Wang, Binghao Zhao, Wenlin Chen, Yu Wang, Wenbin Ma

**Affiliations:** ^1^ Departments of Neurosurgery Peking Union Medical College Hospital Chinese Academy of Medical Sciences and Peking Union Medical College Beijing China

**Keywords:** alternative splicing, low‐grade glioma, nomogram, prediction model

## Abstract

**Background:**

The prognosis of lower‐grade glioma (LGG) differs from that of other grades gliomas. Although lots of studies on the prognostic biomarkers of LGG have been reported, few have significant clinical impact. Alternative splicing (AS) events can affect cell function by splicing precursor mRNA. Therefore, a prognostic model for LGG based on AS events are important to establish.

**Methods:**

RNA sequencing, clinical, and AS event data of 510 LGG patients from the TCGA database were downloaded. Univariate Cox regression analysis was used to screen out prognostic‐related AS events and LASSO regression and multivariate Cox regression were used to establish prognostic risk scores for patients in the training set (*n* = 340). After validation, a nomogram model was established based on the AS signature and clinical information, which was able to predict 1‐, 3‐, and 5‐year survival rates. Finally, considering the regulatory effect of splicing factors (SFs) on AS events, an AS‐SF regulatory network was analyzed.

**Results:**

The most common AS event was exon skipping and the least was mutually exclusive exons. All the seven AS events were related to the prognosis of LGG patients, regardless of whether they were separated or considered as a whole event (integrated AS event), and the integrated AS event had the most significant correlation. After further inclusion of clinical indicators, eight factors were screened out: age, new event, KPS, WHO grade, treatment, integrated AS signature, IDH1 and TP53 mutation status, and a nomogram model was established. The study also constructed an AS‐SF regulatory network.

**Conclusion:**

The AS events and clinical factors that can predict the prognosis of LGG patients were screened, and a prognostic prediction model was established. The results of this study can play an important role in clinical work to better evaluate the prognosis of patients and impact treatment options.

## Graphical abstract

This research analyzed the AS events of lower‐grade glioma patients in TCGA database and figured out some prognostic ones. Then we established a nomogram model based on AS signatures and clinical factors to better predict the survival of patients.

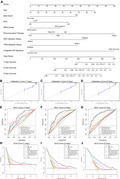

## 1 INTRODUCTION

Glioma, the most common primary intracranial malignant tumor, can be classified into four grades according to the World Health Organization (WHO), with diffuse lower‐grade glioma (LGG) including grades II and III.^1^ The clinical characteristics of LGG patients vary, and the overall survival (OS) and progression‐free survival (PFS) are significantly different, so neither clinical workers nor patients can predict the prognosis.^2^ Numerous studies have been conducted on the prognostic biomarkers of LGG patients. These studies have included not only the well‐known IDH1 mutation status, but also some factors discovered in recent years by bioinformatics analysis, such as eukaryotic initiation factor and some N^6^‐methyladenosine (m^6^A) RNA methylation regulators^3^, ^4^; however, according to the 2016 WHO brain tumor classification, IDH1 mutation status and 1p/19q are the only universally recognized significant prognostic biomarkers of LGG.^5^ Therefore, biomarkers with predictive value for the prognosis of LGG patients are still necessary to better guide the follow‐up review and adjuvant therapy of these patients.

Splicing introns from precursor mRNA, preserving only exons and forming mature mRNA, are necessary steps in intracellular translation. This process plays a crucial role in gene expression.^6^ AS events entrust the diversity of the cellular proteome and influence the biological characteristics of cells in this way.^7^ SFs, which are proteins involved in the regulation of AS, also play an important role in the process of splicing by influencing AS.^8^ Recent studies have shown that AS, due to its influence on gene mutations and expression levels, may cause tumor cell proliferation by affecting oncogenes and inhibiting tumor cell apoptosis.^9^, ^10^ In addition, AS plays a role in drug resistance and tumor metastasis.^11^, ^12^

Considering the broad implications of AS, an increasing number of studies have explored AS events as a prognostic biomarker for cancer patients. In recent years, with the establishment of a tumor database, the bioinformatics analysis between AS events and the outcome of cancer patients has also become a research hotspot.^13^ Although this research continues to emerge, an effective and practical prediction model for LGG patient prognosis based on AS events has not yet been established. Therefore, in this study, bioinformatics analysis technology was used to comprehensively and deeply analyze the clinical information and Splice‐Seq information of LGG patients in The Cancer Genome Atlas (TCGA, https://portal.gdc.cancer.gov/) database, and a prognostic prediction model was established to better serve both clinical work and the patients themselves.

## 2 METHODS

### 2.1 Data inclusion and processing

**Figure 1:**
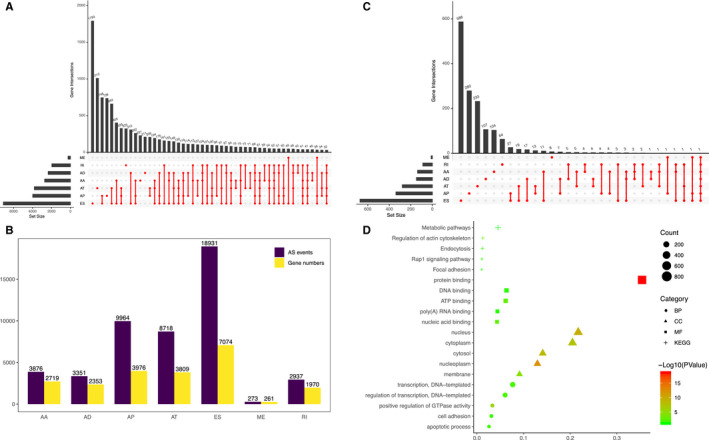
Landscape of alternative splicing (AS) events in lower‐grade glioma (LGG) patients. (A) The distribution of the seven AS events shown in the UpSet plot. (B) Seven types of AS events and the number of their source genes. (C) An UpSet plot to show the prognostic AS events in LGG patients. (D) Functional enrichment analysis of source genes corresponding to survival‐related AS events

This study downloaded the RNA sequencing results and clinical data of 529 patients with LGG in the TCGA (https://portal.gdc.cancer.gov/) and in the Splice‐Seq section of the same database. Information on the AS events of these patients was also downloaded, including the percent‐sliced‐in (PSI) score. This score ranges from 0 to 1, is often used for evaluating AS events, and describes the existence of AS junctions in the exons of the clinical samples we downloaded.^14^ This study used this score to screen all AS events and chose seven of them, including alternate acceptor site (AA), exon skipping (ES), alternate donor site (AD), alternative promoter (AP), mutually exclusive exons (ME), alternate terminator (AT), and retained intron (RI; Figure 1A).

The inclusion criteria of the clinical samples were as follows: (a) age ≥ 18 years old; (b) patients were diagnosed with LGG; (c) RNA‐Seq data could be obtained; (d) splice‐Seq data could be obtained; (e) complete clinical information was available. After screening all 529 patients according to the above inclusion criteria, 510 LGG patients were finally enrolled.

### 2.2 Constructing a prognostic prediction model based on AS events

First, we divided the data of the above 510 patients into a training set (*n* = 340) and a validation set (*n* = 170) according to a 2:1 ratio randomly. Their Clinicopathological characteristics are shown in Table 1. Then, to clarify the correlation between the screened AS events and the survival of LGG patients, we used univariate Cox regression analysis to analyze the PSI score of AS events and the OS of patients using R (Ver 3.5.1). Then, we used the Database for Annotation, Visualization, and Integrated Discovery (DAVID) to complete the functional annotation and pathway analysis for the genes that corresponded to the above AS events. The tools used in the above analysis were Gene Ontology (GO) and Kyoto Encyclopedia of Genes and Genomes (KEGG) pathway analyses, and *p* < 0.05 was regarded as statistically significant (Table S1).

**Table 1:** Clinicopathological characteristics of the TCGA LGG patients in the training set (*n* = 340) and internal validation set (*n* = 170)

Variables	Training set (*n* = 340)	Validation set (*n* = 170)	*p* value
Age (years)	42.9 ± 13.4	42.4 ± 13.1	0.511
Sex			0.166
Female	158	68	
Male	182	102	
New event			0.414
None or NA	151	82	
Yes	189	88	
KPS			0.593
<80	25	15	
≥80	172	91	
NA	143	64	
Histology			0.737
Astrocytoma	127	67	
Oligoastrocytoma	85	45	
Oligodendroglioma	128	58	
WHO grade			0.754
Grade II	163	84	
Grade III	177	86	
Pharmaceutical therapy			0.779
TMZ	155	82	
PCV	22	8	
TMZ + PCV	3	2	
Others	11	3	
NA	149	75	
Radiation therapy			0.843
No	113	53	
Yes	184	93	
NA	43	24	
IDH mutation status			0.764
Wild type	78	37	
Mutant	262	133	
TP53 mutation status			0.060
Wildtype	166	98	
Mutant	174	72	
PTEN mutation status			0.887
Wildtype	323	161	
Mutant	17	9	
EGFR mutation status			0.091
Wildtype	320	153	
Mutant	20	17	
ATRX mutation status			0.699
Wildtype	208	107	
Mutant	132	63	

Note

“New event” included progression and recurrence. “Others” in pharmaceutical therapy included CT + TMT, CT + TMT + Immunotherapy, and CT + Immunotherapy.

Abbreviations: CT, chemotherapy; KPS, Karnofsky performance score; LGG, lower‐grade glioma; NA, not available; PCV, procarbazine, lomustine (CCNU), and vincristine; TMT, targeted molecular therapy; TMZ, temozolomide.

After the univariate Cox regression analysis, we further screened prognostic AS events using least absolute shrinkage and selection operator (LASSO) regression and multivariate Cox regression, and a prognostic scoring formula was established for these AS events as follows: risk score = PSI value of AS event_1_ × β_1_ + PSI value of AS event_2_ × β_2_ + PSI value of AS event_3_ × β_3_ … + PSI value of AS event*_n_* × β*_n_*_._ In this formula, β represents the regression coefficient in the multivariate Cox regression analysis.

To verify the clinical value of the prediction model, we first scored 340 patients in the training set and divided them into high‐ and low‐risk groups according to the scores, with the median of the score as the boundary. Then, Kaplan–Meier (K–M) survival curve analysis was used to analyze the survival of these two groups. The difference in survival between the high‐ and low‐risk groups was analyzed by a two‐sided log‐rank test. Then, we used Harrell's concordance index (C‐index) and time‐dependent receiver operating characteristic (ROC) curve analysis to verify the accuracy and effectiveness of the AS signature prognostic prediction. In addition, to solve the problem of whether AS events are an independent influencing factor, we included all clinical information and carried out a multivariate Cox proportional hazards regression analysis for all factors. Finally, to make the conclusions more rigorous, we performed a further validation using a similar method in the validation set (*n* = 170). Considering that in different LGG patients different biomarkers may have a greater impact on the prognosis, we also conducted a subgroup analysis of the patients.

To ensure that the results of this study would be meaningful for clinical work, we further included the clinical information of patients in the TCGA database, performed univariate Cox analysis, and finally, screened out the factors that have prognostic significance for patients.

### 2.3 Establishing a nomogram of the prognostic model according to AS events and clinical information

The clinical information of the LGG patients, including age, sex, new event (including progression and recurrence), Karnofsky performance status (KPS) score, surgery, pharmaceutical therapy, radiotherapy, IDH1 mutation status, TP53 mutation status, the patients' AS signature, and integrated AS signature, which means considering AS events as a whole for the analysis, were included in the multivariate Cox regression analysis. After this analysis, we established the nomogram scoring system by using all the independent prognostic influencing factors, aiming to form a scoring system capable of evaluating the patients' 1‐, 3‐, and 5‐year OS. To demonstrate the effectiveness of the system, its discrimination performance was evaluated using the C‐index, area under the ROC curve (AUC) and calibration plots.^15^, ^16^ The clinical applicability of this scoring system was evaluated using decision curve analysis.^17^ Finally, to verify the effectiveness of the prognostic prediction model, we used the validation set (*n* = 170) to perform analyses including C‐index, AUC, and calibration plots. All the above analyses were conducted using R (version 3.5.1), and a *p* < 0.05 was considered statistically significant.

### 2.4 Correlation analysis between AS events and SFs and the establishment of a regulatory network among them

In the tumor microenvironment, SFs can regulate AS events, therefore, the establishment of a prognostic prediction model based on AS events needs to explore the relationship between SFs and AS events. First, Pearson's correlation test was used to analyze the PSI value of AS events and the expression level of SFs that could regulate these AS events. *P* < 0.001 and correlation coefficients >0.6 or < −0.6 were considered to be statistically significant. Then, based on the analysis results, we established a regulatory network between AS events and SFs and visualized the regulatory network using Cytoscape.

## 3 RESULTS

### 3.1 Distribution of AS events in LGG patients in the TCGA

By analyzing the Splice‐Seq data of 510 LGG patients in the TCGA database, we obtained a total of 48,050 AS events and 22,162 corresponding genes. Among them were 3876 AA events and 2719 corresponding genes; 3351 AD events and 2353 corresponding genes; 9964 AP events and 3976 corresponding genes; 8718 AT events and 3809 corresponding genes; 18,931 ES events and 7074 corresponding genes; 273 ME events and 261 corresponding genes; and 2937 RI events and 1907 corresponding genes (Figure 1B). Among all AS events, ES was the most common, accounting for 39.40%. The second most common was AP and the least common was ME.

### 3.2 Screening of prognostic AS events in LGG patients and functional annotation of corresponding genes

After completing a univariate Cox regression analysis of all AS events, we screened 2,185 prognostic AS events and 1,520 corresponding source genes (Figure 1C). In the corresponding source genes, we conducted a GO analysis, which included biological processes (BPs), cellular components (CCs), and molecular functions (MFs). According to the BP results, these genes were mainly enriched in transcription and the regulation of transcription. The results of the CC analysis showed that these genes were mainly enriched in the nucleus and cytoplasm. The MF results indicated that these genes were enriched in protein, DNA and ATP binding (Figure 1D).

**Figure 2:**
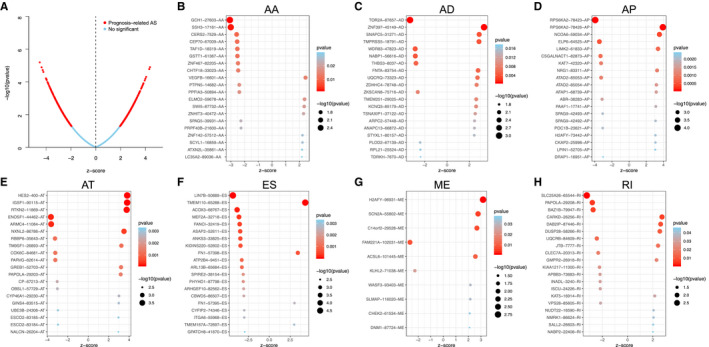
Prognostic alternative splicing (AS) events in lower‐grade glioma (LGG) patients. (A) A volcano plot shows the overview of prognostic AS events. (B–H) Bubble plots show the top 20 prognostic AS events (*z*‐score > 0, HR > 1; *z*‐score < 0, HR < 1)

According to the hazard ratio (HR) or *z*‐score, AS events that are predictive of patient prognosis were divided into two categories: favorable prognostic factors (HR < 1 or *z*‐score < 0) and unfavorable prognostic factors (HR > 1 or *z*‐score > 0). Figure 2A–H shows the top 20 AS events of all 7 AS events, with most being favorable events (1247 vs. 938).

### 3.3 Establishment and validation of the prognostic risk score model based on AS events

**Figure 3:**
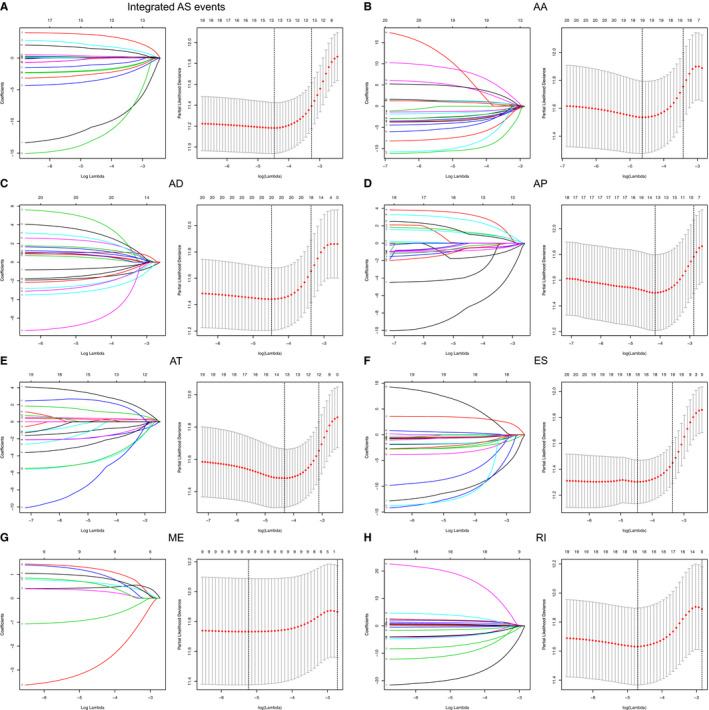
Alternative splicing (AS) events related to the prognosis of lower‐grade glioma (LGG) patients were screened and verified with LASSO regression. The left panel is a coefficient profile plot. A vertical line is shown at the value that was selected by 10‐fold cross‐validation. The optimal log produced 10 characteristics of nonzero coefficients. The right panel depicts the process of selecting optimal parameters by LASSO regression using 10‐fold cross‐validation. We plotted the partial likelihood deviance curve with log, and a dotted vertical line is shown at the optimal values. This process was accomplished using the 1 standard error of the minimum and maximum criteria

**Figure 4:**
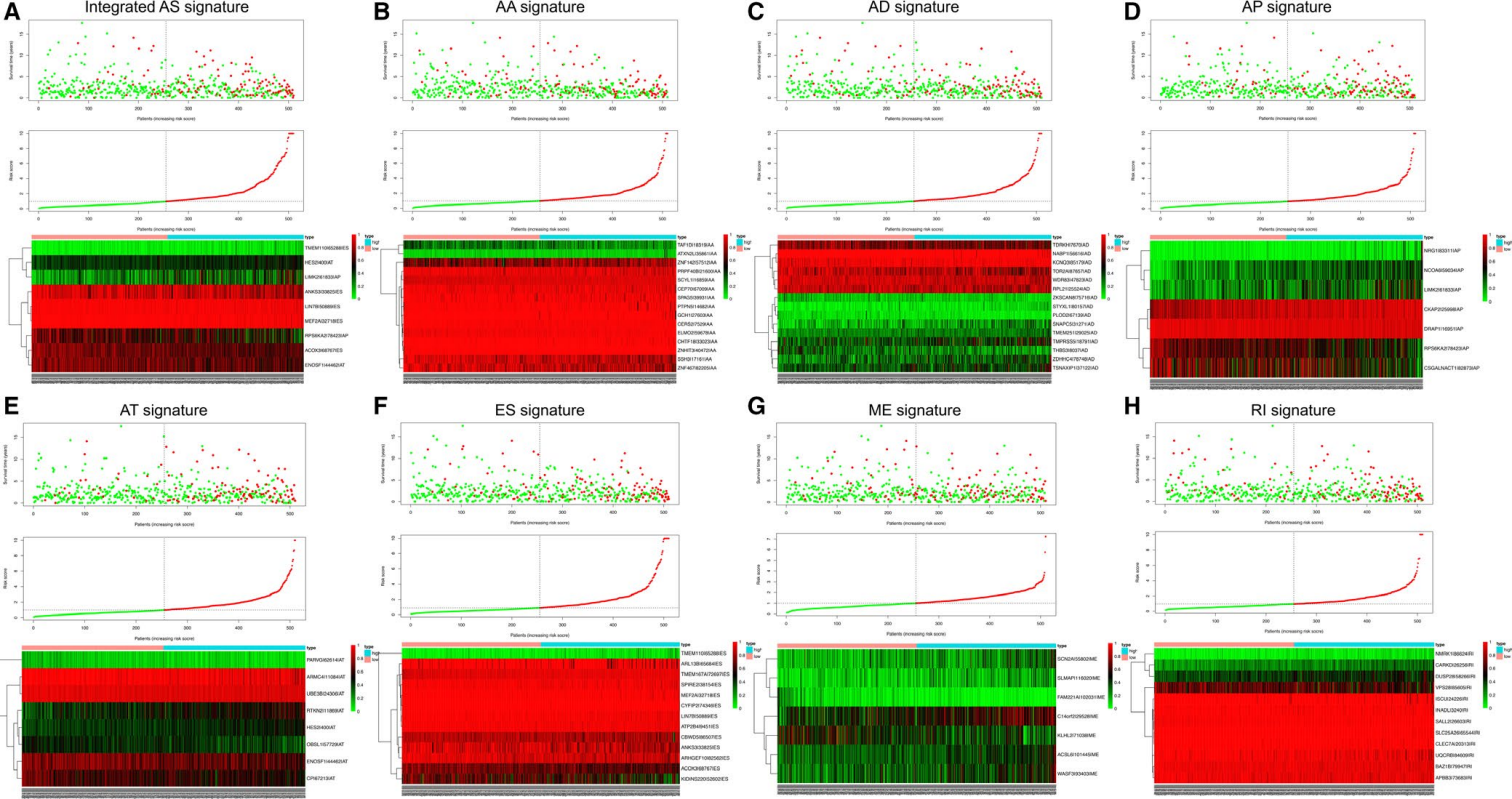
We analyzed the risk scores of seven types of alternative splicing (AS) signatures and integrated AS signatures in lower‐grade glioma (LGG) patients. The upper panel shows the distribution of the survival time of patients with different risk scores. The middle panel figure shows the variation trend of patient survival time with risk scores. The bottom panel shows the heat map of survival‐related AS events. The red and green in the figure represent high‐ and low‐expression levels, respectively. The patients were divided into high‐ and low‐risk groups according to the relationship between the risk score and survival time

**Figure 5:**
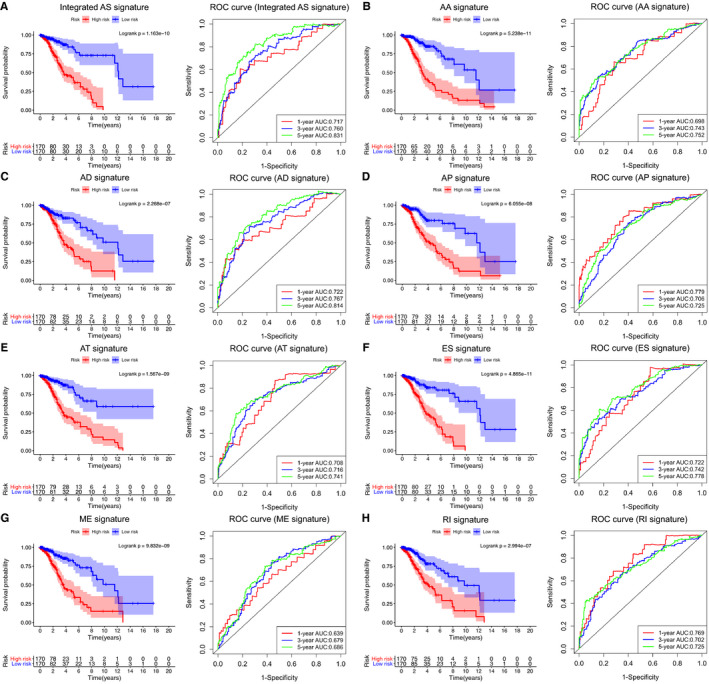
Survival analysis of the patients based on the alternative splicing (AS) signature and validation of the prognostic performance of all AS signatures and integrated AS signature in the training set (*n* = 340). The left panel shows the K–M survival curve of the patients in the high‐ and low‐risk groups. The right panel shows the ROC curve demonstrating the prognostic performance of each AS signature and integrated AS signature

We used the above seven AS events and integrated the AS events to further carry out LASSO regression and multivariate Cox regression analyses based on the results of the univariate Cox regression analysis (Figure 3A–H). As shown in Table 2, we established prognostic risk score formulas for the seven AS events and integrated the AS events (Table S2), and these formulas were used to score each LGG patient in our study. Then, with the median‐risk score as the boundary, 340 patients in the training set were divided into the high‐risk group (score higher than the median‐risk score) and the low‐risk group (score lower than the median‐risk score; Figure 4A–H). For the two groups of patients, a K‐M survival curve analysis was conducted, which showed that the OS of the eight groups of patients in the high‐risk group was significantly shorter than the low‐risk group (*p* < 0.05, Figure 5A–H). Among them, the integrated AS signature had the highest degree of differentiation for OS, with a C‐index of 0.791 (95% CI, 0.752–0.830; *p* = 1.54 × 10^−17^), which indicates that this score plays the most significant role in all AS‐based risk scores (Table 2). Although the impact of the integrated AS signature was the most significant among all AS signatures, the remaining seven signatures also showed a good predictive effect on patients' 1‐, 3‐, and 5‐year OS rates after being analyzed by a time‐dependent ROC analysis. The above results demonstrate the importance of AS events for LGG patients.

**Table 2:** The prognostic risk score models based on the PSI values of AS event types of the LGG training set (*n* = 340)

AS event types	Formula (risk score model)	C‐index (95% CI)	*p* value
Integrated AS signature	LIN7B‐50889‐ES × (−12.74) + TMEM110‐65288‐ES × 4.29 + RPS6KA2‐78423‐AP × (−2.35) + ACOX3‐68767‐ES × (−4.42) + HES2‐400‐AT × 2.33 + ENOSF1‐44462‐AT × (−3.06) + MEF2A‐32718‐ES × (−15.58) + ANKS3‐33825‐ES × (−2.41) + LIMK2‐61833‐AP × 1.85	0.791 (0.752–0.830)	1.54 × 10^−17^
AA signature	GCH1‐27603‐AA × (−4.03) + SSH3‐17161‐AA × (−3.70) + CERS2‐7529‐AA × (−16.89) + CEP70‐67009‐AA × (−4.25) + TAF1D‐18319‐AA × (−2.15) + ZNF467‐82205‐AA × (−2.86) + CHTF18‐33023‐AA × (−8.09) + PTPN5‐14682‐AA × (−5.85) + ELMO2‐59678‐AA × 10.57 + ZNHIT3‐40472‐AA × 18.52 + SPAG5‐39931‐AA × (−3.54) + PRPF40B‐21600‐AA × (−3.84) + ZNF142‐57512‐AA × 1.65 + SCYL1‐16859‐AA × 6.96 + ATXN2L‐35861‐AA × 5.83	0.739 (0.699–0.778)	4.88 × 10^−16^
AD signature	TOR2A‐87657‐AD × (−2.36) + SNAPC5‐31271‐AD × 2.06 + TMPRSS5‐18791‐AD × 1.40 + WDR83‐47823‐AD × (−3.64) + NABP1‐56616‐AD × (−7.22) + THBS3‐8037‐AD × (−1.14) + ZDHHC4‐78748‐AD × 1.75 + ZKSCAN8‐75716‐AD × (−2.53) + TMEM251‐29025‐AD × 3.20 + KCNQ3‐85179‐AD × 4.58 + TSNAXIP1‐37122‐AD × 1.06 + STYXL1‐80157‐AD × 3.67 + PLOD2‐67139‐AD × (−3.40) + RPL21‐25524‐AD × (−2.02) + TDRKH‐7670‐AD × (−1.94)	0.739 (0.685–0.794)	7.92 × 10^−18^
AP signature	RPS6KA2‐78423‐AP × (−2.16) + NCOA6‐59034‐AP × 1.71 + LIMK2‐61833‐AP × 1.61 + CSGALNACT1‐82873‐AP × (−1.07) + NRG1‐83311‐AP × 4.44 + CKAP2‐25998‐AP × 3.89 + DRAP1‐16951‐AP × (−9.90)	0.727 (0.675–0.778)	2.32 × 10^−17^
AT signature	HES2‐400‐AT × 4.00 + RTKN2‐11869‐AT × 1.62 + ENOSF1‐44462‐AT × (−1.99) + ARMC4‐11084‐AT × (−5.67) + PARVG‐62614‐AT × (−5.35) + CP−67213‐AT × (−2.58) + OBSL1‐57729‐AT × (−3.05) + UBE3B‐24306‐AT × (−8.60)	0.697 (0.642–0.753)	4.35 × 10^−12^
ES signature	LIN7B‐50889‐ES × (−14.51) + TMEM110‐65288‐ES × 3.66 + ACOX3‐68767‐ES × (−2.95) + MEF2A‐32718‐ES × (−13.74) + ANKS3‐33825‐ES × (−1.93) + KIDINS220‐52602‐ES × (−0.87) + ATP2B4‐9451‐ES × (−10.29) + ARL13B‐65684‐ES × (−2.03) + SPIRE2‐38154‐ES × (−5.10) + ARHGEF10‐82562‐ES × (−2.94) + CBWD5‐86507‐ES × (−3.01) + CYFIP2‐74346‐ES × (−14.51) + TMEM167A‐72697‐ES × 10.70	0.735 (0.686–0.785)	1.81 × 10^−20^
ME signature	ACSL6‐101445‐ME × 1.46 + KLHL2‐71038‐ME × (−1.24) + SLMAP‐116020‐ME × 1.41 + C14orf2‐29528‐ME × 0.93 + SCN2A‐55802‐ME × 1.20 + FAM221A‐102031‐ME × (−3.97) + WASF3‐93403‐ME × 1.02	0.681 (0.642–0.720)	2.32 × 10^−8^
RI signature	SLC25A26‐65544‐RI × (−20.42) + BAZ1B‐79947‐RI × (−8.98) + CARKD‐26256‐RI × 2.59 + DUSP28‐58266‐RI × 1.94 + UQCRB‐84609‐RI × (−4.45) + CLEC7A‐20313‐RI × (−13.52) + APBB3‐73683‐RI × (−5.29) + INADL‐3240‐RI × (−4.11) + ISCU‐24226‐RI × (−4.40) + VPS28‐85605‐RI × (−1.98) + NMRK1‐86624‐RI × 4.32 + SALL2‐26603‐RI × 22.37	0.727 (0.679–0.775)	2.01 × 10^−20^

Abbreviations: AA, alternate acceptor site; AD, alternate donor site; AP, alternate promoter; AS, alternative splicing; AT, alternate terminator; CI, confidence interval; ES, exon skip; LGG, lower‐grade glioma; ME, mutually exclusive exons; PSI, percent‐spliced‐in; RI, retained intron.

Finally, to verify that AS events have a significant impact on patient survival, we used a similar method for verification in the validation set (*n* = 170), and further determined the prognostic value of the AS signatures (Figure S1). Considering that some important biomarkers such as IDH may have a greater impact on the survival of LGG patients, we divided the patients into different subgroups based on biomarkers and analyzed the survival in the different subgroups. The results showed that the integrated AS signature still has the most significant predictive effect on the survival of patients in different subgroups (Figure S2).

### 3.4 The AS signature and clinical factors are independent prognostic factors of LGG patients

After demonstrating the predictive effect of the above eight AS signatures on prognosis, we further included various clinical information of the patients and analyzed them together with the AS signature (Table 3). Univariate Cox analysis showed that patient age (*p* < 0.001), new event (*p* = 0.001), KPS score (*p* = 0.001), WHO grade (*p* < 0.001), pharmaceutical treatment (*p* < 0.001), IDH1 mutation status (*p* < 0.001), TP53 mutation status (*p* < 0.001), and all AS signatures (*p* < 0.001) were significantly correlated with patient OS.

**Table 3:** Univariate and multivariate cox proportional hazards analysis of clinical parameters and AS event‐based risk score model of the TCGA LGG patients in the training set (*n* = 340) and internal validation set (*n* = 170)

Variables	Training set (*n* = 340)				Validation set (*n* = 170)			
	Univariate analysis		Multivariate analysis		Univariate analysis		Multivariate analysis	
	HR (95% CI)	*p*	HR (95% CI)	*p*	HR (95% CI)	*p*	HR (95% CI)	*p*
Age	1.03 (1.02–1.04)	**5.67 × 10^−6^**	1.02 (1.01–1.04)	**2.44 × 10^−3^**	1.03 (1.02–1.05)	**3.13 × 10^−5^**	1.03 (1.01–1.05)	**4.18 × 10^−3^**
Sex (Female/Male)	0.90 (0.63–1.27)	0.54	—	—	0.88 (0.58–1.34)	0.53	—	—
New event (None or NA/Yes)	2.11 (1.35–3.30)	**1.08 × 10^−3^**	1.65 (1.03–2.63)	**3.57 × 10^−2^**	2.07 (1.20–3.57)	**8.92 × 10^−3^**	1.70 (1.66–1.74)	**1.03 × 10^−2^**
KPS (<80/ ≥ 80/NA)	0.61 (0.45–0.82)	**1.25 × 10^−3^**	0.84 (0.80–0.88)	**3.05 × 10^−5^**	0.62 (0.47–0.88)	**8.27 × 10^−3^**	0.84 (0.79–0.88)	**3.86 × 10^−2^**
Histology (Astrocytoma/Oligoastrocytoma/Oligodendroglioma)	0.98 (0.80–1.20)	0.83	—	—	1.10 (0.87–1.40)	0.43	—	—
WHO grade (Grade II/III)	2.15 (1.48–3.11)	**5.96 × 10^−5^**	1.46 (1.07–1.85)	**7.53 × 10^−3^**	2.24 (1.43–3.50)	**3.96 × 10^−4^**	1.74 (1.05–2.93)	**3.18 × 10^−3^**
Pharmaceutical therapy (TMZ/PCV/TMZ + PCV/Others/NA)	0.84 (0.80–0.88)	**1.23 × 10^−6^**	0.71 (0.67–0.75)	**8.90 × 10^−3^**	0.82 (0.76–0.87)	**1.47 × 10^−2^**	1.09 (1.05–1.13)	**2.43 × 10^−2^**
Radiation therapy (No/Yes/NA)	1.13 (0.84–1.52)	0.42	—	—	1.01 (0.72–1.44)	0.94	—	—
Surgery (Biopsy only/Tumor resection)	0.82 (0.59–1.44)	0.77	—	—	0.93 (0.54–1.32)	0.81	—	—
IDH1 mutation status (Wildtype/Mutant)	0.40 (0.28–0.57)	**3.85 × 10^−7^**	0.64 (0.44–0.94)	**2.17 × 10^−2^**	0.37 (0.24–0.56)	**3.90 × 10^−6^**	0.70 (0.66–0.74)	**1.58 × 10^−2^**
TP53 mutation status (Wildtype/Mutant)	2.01 (1.39–2.92)	**2.13 × 10^−4^**	1.46 (1.07–1.86)	**5.95 × 10^−3^**	2.03 (1.30–3.17)	**1.85 × 10^−3^**	1.85 (1.12–3.06)	**1.63 × 10^−2^**
PTEN mutation status (Wildtype/Mutant)	1.27 (0.59–2.74)	0.53	—	—	1.95 (0.90–4.22)	0.09	—	—
EGFR mutation status (Wildtype/Mutant)	0.88 (0.46–1.68)	0.69	—	—	1.27 (0.61–2.63)	0.53	—	—
ATRX mutation status (Wildtype/Mutant)	0.85 (0.59–1.23)	0.39	—	—	0.75 (0.48–1.18)	0.21	—	—
Integrated AS signature (Low‐risk score/High‐risk score)	4.81 (3.06–7.56)	**8.76 × 10^−12^**	1.99 (1.32–3.02)	**3.63 × 10^−9^**	5.23 (2.99–9.13)	**5.81 × 10^−9^**	2.91 (1.72–4.94)	**7.03 × 10^−5^**
AA signature (Low‐risk score/High‐risk score)	3.71 (2.51–5.48)	**4.48 × 10^−11^**	1.30 (0.74–2.26)	0.36	4.40 (2.72–7.11)	**1.55 × 10^−9^**	1.30 (0.63–2.66)	0.47
AD signature (Low‐risk score/High‐risk score)	3.45 (2.32–5.13)	**9.20 × 10^−10^**	1.16 (0.77–1.55)	0.096	3.29 (2.05–5.28)	**8.67 × 10^−7^**	1.37 (0.76–2.46)	0.30
AP signature (Low‐risk score/High‐risk score)	3.66 (2.44–5.50)	**4.21 × 10^−10^**	1.06 (0.67–1.45)	0.26	3.55 (2.18–5.79)	**3.56 × 10^−7^**	1.49 (0.82–2.70)	0.19
AT signature (Low‐risk score/High‐risk score)	4.28 (2.74–6.69)	**1.64 × 10^−10^**	1.50 (0.89–2.51)	0.13	4.24 (2.55–7.05)	**2.80 × 10^−8^**	1.37 (0.75–2.49)	0.30
ES signature (Low‐risk score/High‐risk score)	4.05 (2.64–6.20)	**1.45 × 10^−10^**	1.05 (0.65–1.44)	0.32	5.28 (3.06–9.12)	**2.27 × 10^−9^**	2.26 (0.88–4.54)	0.21
ME signature (Low‐risk score/High‐risk score)	2.68 (1.84–3.92)	**3.34 × 10^−7^**	1.09 (0.71–1.66)	0.69	3.68 (2.29–5.92)	**7.08 × 10^−8^**	1.43 (0.81–2.51)	0.22
RI signature (Low‐risk score/High‐risk score)	3.14 (2.12–4.66)	**1.26 × 10^−8^**	1.29 (0.81–2.06)	0.28	3.14 (1.98–4.98)	**1.08 × 10^−6^**	1.10 (0.63–1.91)	0.74

Note

“New event” included progression and recurrence. “Others” in pharmaceutical therapy included CT + TMT, CT + TMT + Immunotherapy, and CT + Immunotherapy. All statistical tests were two‐sided.

Bold numbers indicate that the P value is significant, which means that the corresponding factors are associated with the outcome of patients significantly.

Abbreviations: AA, alternate acceptor site; AD, alternate donor site; AP, alternate promoter; AS, alternative splicing; AT, alternate terminator; CI, confidence interval; CT, chemotherapy; ES, exon skip; HR, hazard ratio; KPS, Karnofsky performance score; LGG, lower‐grade glioma; ME, mutually exclusive exons; NA, not available; PCV, procarbazine, lomustine (CCNU), and vincristine; RI, retained intron; TMT, targeted molecular therapy; TMZ, temozolomide.

Based on the univariate Cox analysis, a multivariate Cox analysis was used to further screen independent factors that could affect the prognosis of patients including age (*p* = 0.002), new event (*p* = 0.036), KPS score (*p* < 0.001), WHO grade (*p* = 0.002), pharmaceutical treatment (*p* = 0.009), IDH1 mutation status (*p* = 0.022), TP53 mutation status (*p* = 0.006), and integrated AS signature (*p* < 0.001).

To further verify the above conclusions, we also analyzed the data of 170 LGG patients in the validation set and screened out the same prognostic prediction factors (Table 3). Therefore, we used the eight factors obtained above to carry out the next series of analyses, and established a nomogram model, which is more practical in clinical work.

### 3.5 Establishment of a nomogram of the prognostic model that included the AS signature

**Figure 6:**
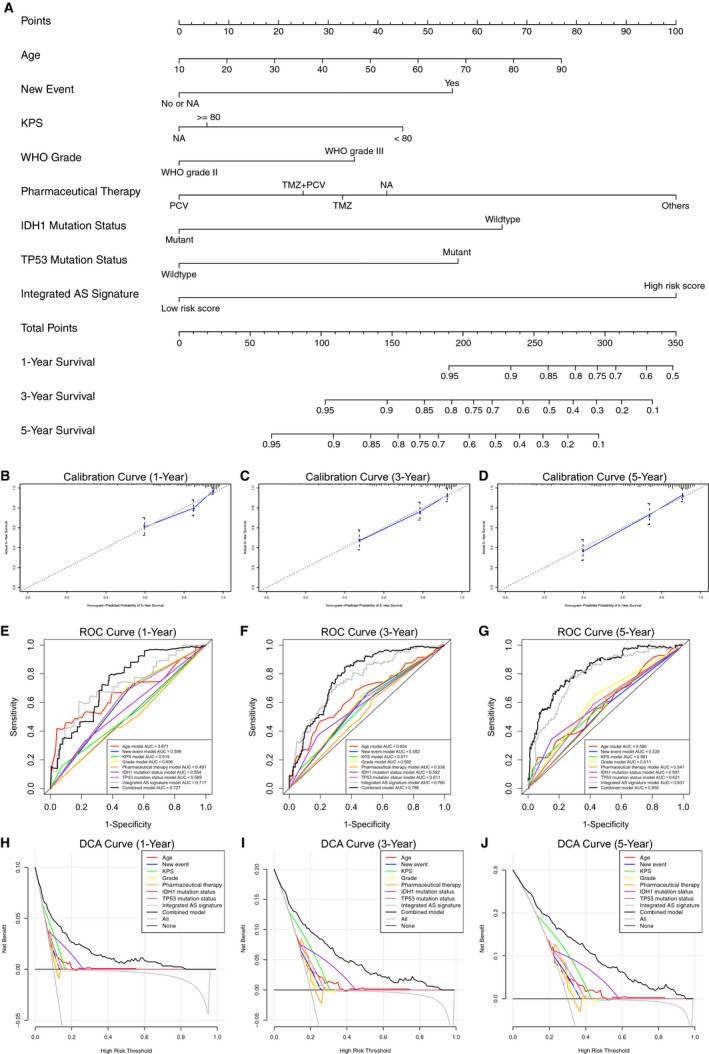
The nomogram of the prognostic prediction model was established based on the alternative splicing (AS) signature and the validation of the model on the predictive efficacy of the survival time in lower‐grade glioma (LGG) patients. (A) The prognostic prediction model established for LGG patients that can predict the 1‐, 3‐, and 5‐year survival. (B–D) A calibration plot to verify the prognostic performance of the model with the predicted value on the *x*‐axis and the actual value on the *y*‐axis. (E–G) A ROC curve to verify the prognostic performance of the model through comparison with other single factors. (H–J) A DCA curve to verify the prognostic performance of the model by comparison with other single factors

To better apply the results of this study to clinical work, we established a nomogram prognostic score system based on the above results to predict the 1‐, 3‐, and 5‐year survival of patients. This scoring system includes age, new event, KPS score, WHO grade, pharmaceutical therapy, IDH1 mutation status, TP53 mutation status, and integrated AS signature (Figure 6A). Then, to verify the reliability of this model, we conducted a calibration plot (Figure 6B–D) and an AUC of the ROC curve analysis (Figure 6E–G) again. The results all verified the practical value of the model. As shown in Figure 6E–G, the prediction effect of a single factor on the prognosis of patients is far less significant than that of a multifactor.

Then, we analyzed the above data again using decline curve analysis (DCA) to verify the ability of the nomogram to predict the 1‐, 3‐, and 5‐year survival of patients (Figure 6H–J). The results showed that the nomogram as a whole was significantly better than individual factors in predicting prognosis. To verify the universality of the nomogram prognostic prediction model, we used the data of 170 LGG patients in the validation set to verify again, and further affirmed the predictive value of the model (Figure S3). Considering that no effective survival prediction model currently exists for LGG patients, the establishment of this model is undoubtedly of great significance to both clinical workers and patients.

### 3.6 Regulatory network analysis between AS events and SFs

**Figure 7:**
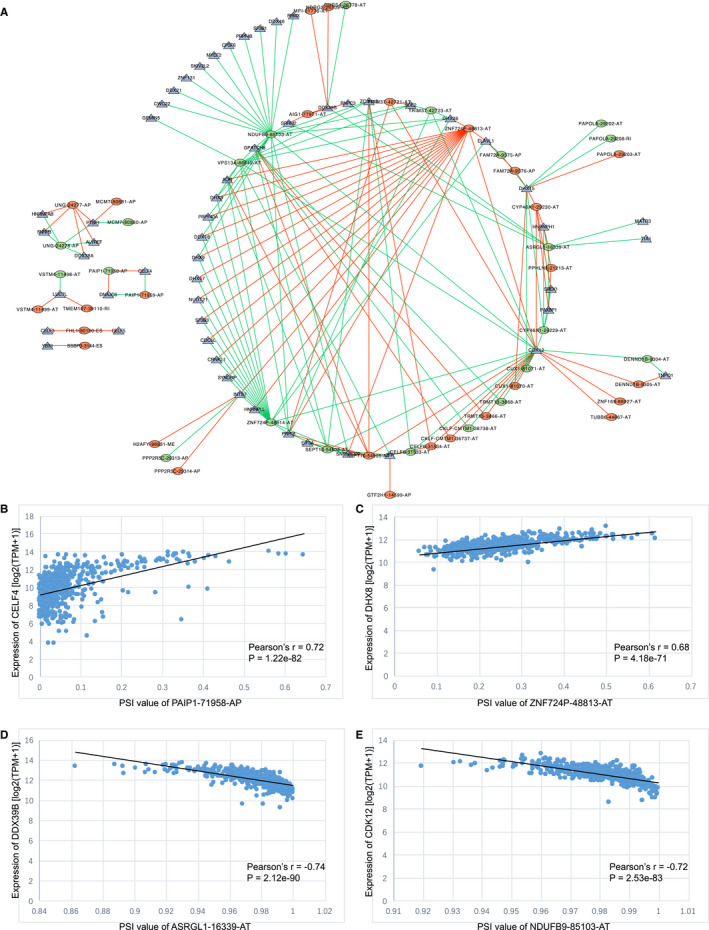
Analysis of the correlation between alternative splicing (AS) events and splicing factors (SFs). (A) The regulatory network between AS events and SFs. The green and red dots in the figure represent the favorable and unfavorable prognostic AS events, respectively; the blue triangle represents the SFs, and the green and red lines represent the negative and positive correlation between AS events and SFs, respectively. (B–C) The two AS‐SF pairs with the strongest positive correlation are CELF4 and PAIP1‐71958‐AP (Pearson correlation coefficient = 0.72) and DHX8 and ZNF724P‐48813‐AT (Pearson correlation coefficient = 0.68). (D–E) The two AS‐SF pairs with the strongest negative correlation are DDX39B and ASRGL1‐16339‐AT (Pearson correlation coefficient = −0.74) and CDK12 and NDUFB9‐85103‐A (Pearson correlation coefficient = −0.72)

By conducting survival and correlation analyses on the AS sequencing data and RNA sequencing expression data, we screened 55 SFs and 48 AS events that were correlated with patient survival (Pearson correlation coefficient >0.6 or < −0.6, *p* < 0.001; Table S3). The above SFs and AS events constitute a total of 173 SF‐AS pairs, of which 73 pairs are positively correlated and 100 pairs are negatively correlated; we constructed these paired SFs and AS events into a regulatory network (Figure 7A) and found that the following four SF‐AS pairs were most significantly correlated with each other: CELF4 and PAIP1‐71958‐AP, DHX8 and ZNF724P‐48813‐AT, DDX39B and ASRGL1‐16339‐AT, and CDK12 and NDUFB9‐85103‐AT (Figure 7B–E).

## 4 DISCUSSION

As one of the factors that plays an important role in the development of tumors, AS events have gradually become a popular research topic for oncologists. Currently, AS events have been studied in lung cancer, prostate cancer, and acute myeloid leukemia. Studies have shown that in lung cancer, after aberrant splicing, the expression of BCL2L1, MDM2, MDM4, NUMB, and MET may play an important role in tumorigenesis, which may be due to the effects on cell apoptosis, cell proliferation, and intercellular cohesion‐related pathways.^18^, ^19^, ^20^ Coomer et al also mentioned the role of SFs in tumorigenesis when they summarized the impact of AS events in lung cancer.^21^ SFs, including QKI, RBM4, RBM5, RBM6, RBM10, and SRSF1, were involved in the development of lung cancer, as SFs play a regulatory role in splicing. Paschalis et al. summarized the effect of AS events in prostate cancer, stating that they can influence tumor development and metastasis by acting on androgen receptors, as well as the effect of hormone therapy and radiotherapy.^22^ Anande et al reported that RNA splicing alterations may be closely related to the prognosis of AML patients, and therefore, serve as a biomarker of this disease.^23^ In glioma, the study of AS events is still in the initial stage, but the relationship between AS events and the prognosis of glioma patients has been analyzed.^13^ Chai et al. proposed a new classification of LGG based on AS events.^24^

The above studies demonstrate that AS events play a crucial role in tumors. Therefore, translating the results of these studies into clinical work has become the purpose of all researchers. One of the highlights of our study is the construction of a prognostic model with a nomogram that uses both clinical factors and AS events, making this model more clinically practical. In recent years, the nomogram of the prognostic model has been developed in a variable subfield of glioma. Gittleman et al. screened the several factors related to patient survival through the analysis of LGG patients in the TCGA database and The Ohio Brain Tumor Study (OBTS) database.^25^ These factors included tumor grade, age of diagnosis, KPS score, and IDH1 mutation status. The authors further analyzed the above factors and constructed a prognostic prediction model using a nomogram. Similarly, Zhao et al. also constructed a prognostic prediction model based on clinical data downloaded from the Surveillance, Epidemiology, and End Result (SEER) database.^26^ New genes related to prognosis have been found based on database analysis, and a prognostic prediction model has been constructed.^27^, ^28^ With the development of radiology in recent years, research on establishing nomogram models in glioma based on radiomics data has also gradually increased, and this includes the preoperative prediction of tumor grade and the prediction of molecular biomarkers.^29^, ^30^ Based on clinical factors and AS events, an epigenetic factor closely related to the genesis, progression, metastasis, and drug resistance of tumors, this study established a nomogram of the prognostic prediction model for LGG patients, demonstrating the value of basic science research and the importance of translational medicine.

The RNA‐Seq data of 510 LGG patients in the TCGA database were analyzed. Univariate Cox analysis was used to screen 2185 AS events related to prognosis, and a prognostic risk score model based on AS events was further established based on multivariate Cox regression and LASSO regression. Although all seven AS Signatures can be related to the prognosis of LGG patients separately, we found that when the seven AS events are considered as a whole, the integrated AS signature could be an independent prognostic factor. Therefore, we used the integrated AS signature and clinical information of the patients to establish a nomogram prognosis prediction model. To further clarify the role of AS Events, the regulatory network between AS events and SFs was analyzed. The prognosis of LGG patients is known to be significantly different, and few remarkable biomarkers are available except for IDH and 1p/19q. This study focused on the AS events that have not been well explored in LGG. A large amount of information from 510 samples was enrolled in the TCGA database for analysis and further verification. The integrated AS signature was significantly correlated with prognosis and could be used as an important biomarker. Based on this, a clinically applicable prediction model was established, which is not only beneficial for the diagnosis and treatment of LGG patients, but also a direction for further exploration.

This study has some limitations. First, as mentioned above, the effect of AS events on RNA is regulated by SFs.^8^ Although this study analyzed the regulatory network between AS events and SFs and found four AS‐SF pairs with the strongest correlation, we did not perform an additional in‐depth analysis of their relationship, which may warrant future research. The second disadvantage is that this study included only well‐known clinical and pathological indicators in LGG patients without exploring other new potential biomarkers due to the consideration of clinical practicability. To make the model more accurate, future studies should be conducted to explore new prognostic factors. Additionally, surgical factors and imaging data could also be considered for analysis. The above two deficiencies can be further studied in the future.

In conclusion, the value of AS events, as a factor closely related to tumorigenesis and development, is fully reflected in glioma. In this study, AS events and clinical data of LGG patients in the TCGA database were analyzed, and prognostic factors were screened. Then, a nomogram of the prognostic prediction model was established based on the factors that can predict the 1‐, 3‐, and 5‐year survival of LGG patients. The establishment of this model will help clinical workers better evaluate the prognosis of patients and will play a role in guiding follow‐up and treatment processes.

## CONFLICTS OF INTEREST

The authors have no conflicts of interest to disclose.

## AUTHOR CONTRIBUTIONS

All authors designed and conducted this article. All authors read and approved the final manuscript. Notably, Yaning Wang and Zihao Wang equally share the first authorship; Yu Wang and Wenbin Ma equally share the corresponding authorship.

## Supporting information

Fig S1

Fig S2

Fig S3

Table S1

Table S2

Table S3

## Data Availability

The data are available upon request of the corresponding authors.
