# Supplementary figures and images for: Development of a nomogram for prognostic prediction of lower‐grade glioma based on alternative splicing signatures
Source: Cancer Med. 2020 Oct 13;9(24):9266–81. doi: 10.1002/cam4.3530 (PMC7774734; doi:10.1002/cam4.3530)

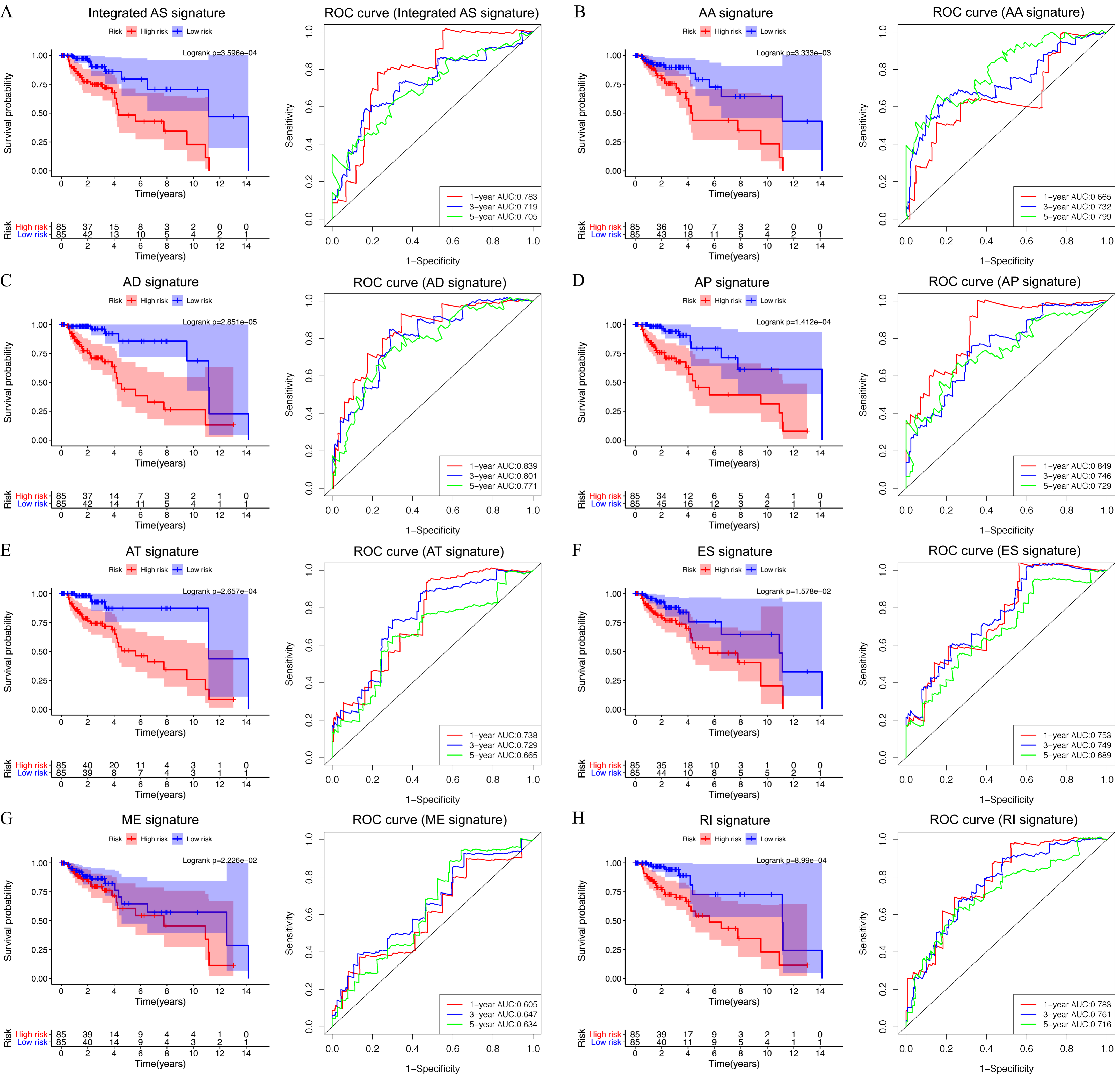

Supplement: Supplementary file 1 — Fig S1 [file CAM4-9-9266-s001.pdf]

A

Training set (N=340)

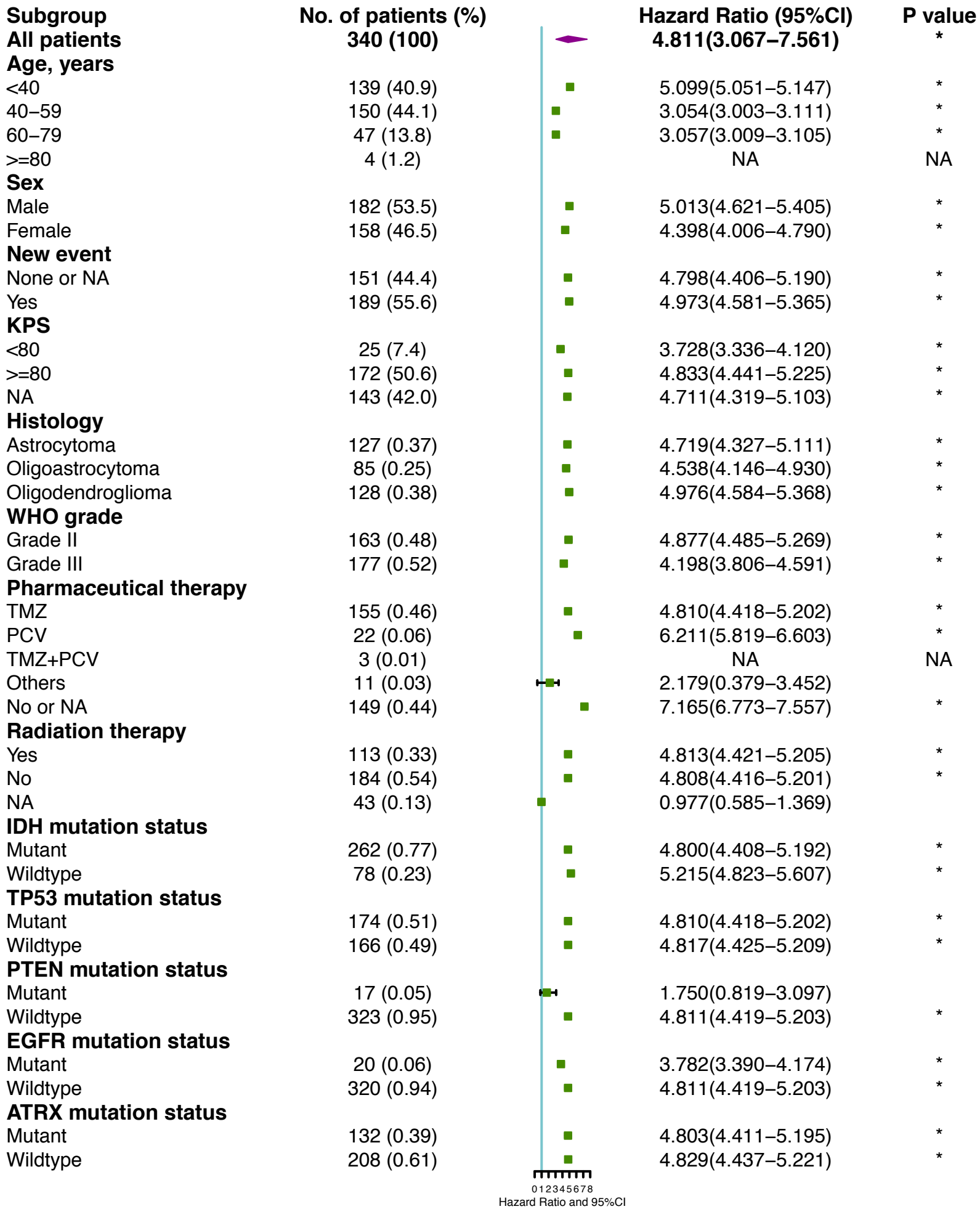

B

Validation set (N=170)

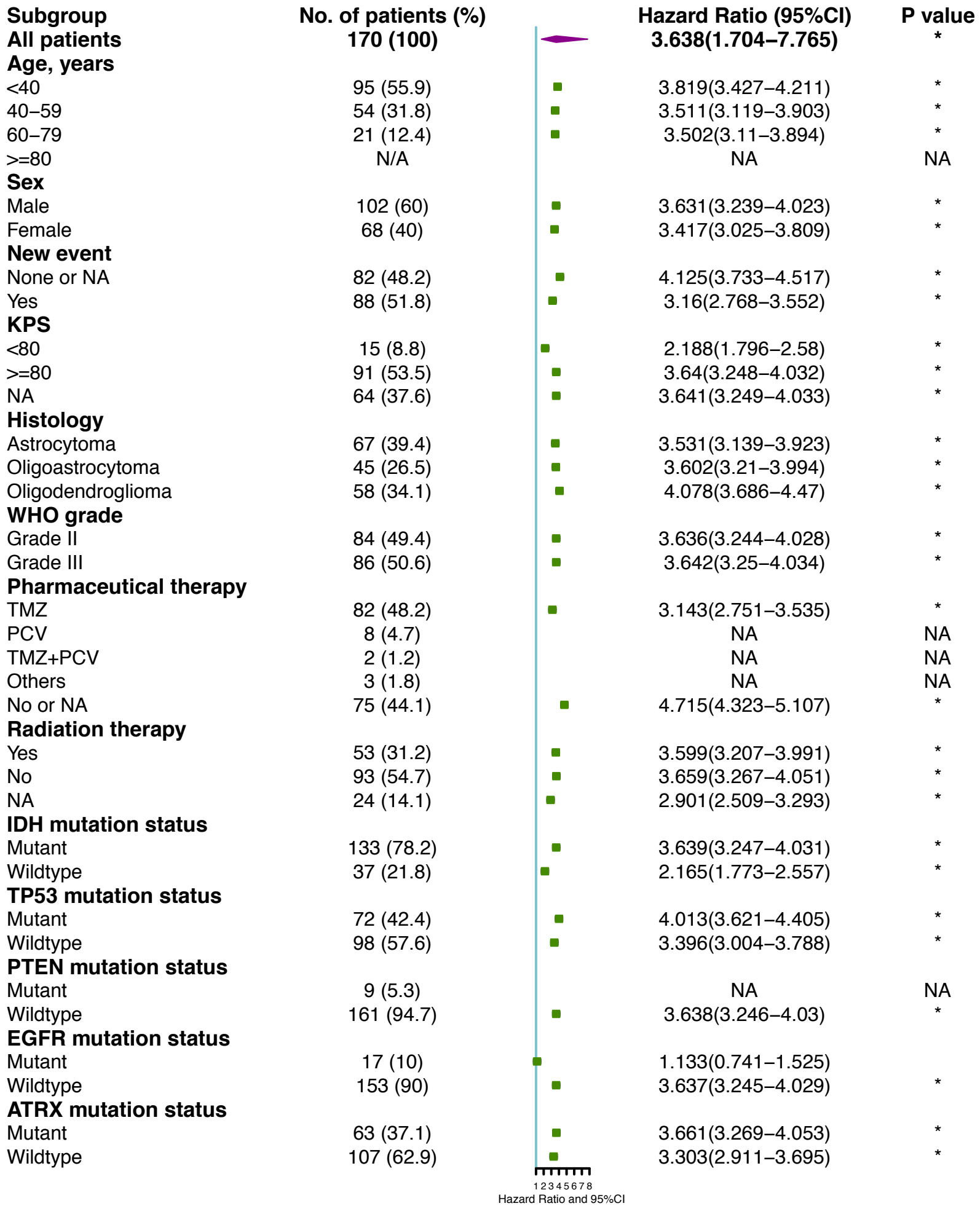

Supplement: Supplementary file 2 — Fig S2 [file CAM4-9-9266-s002.pdf]

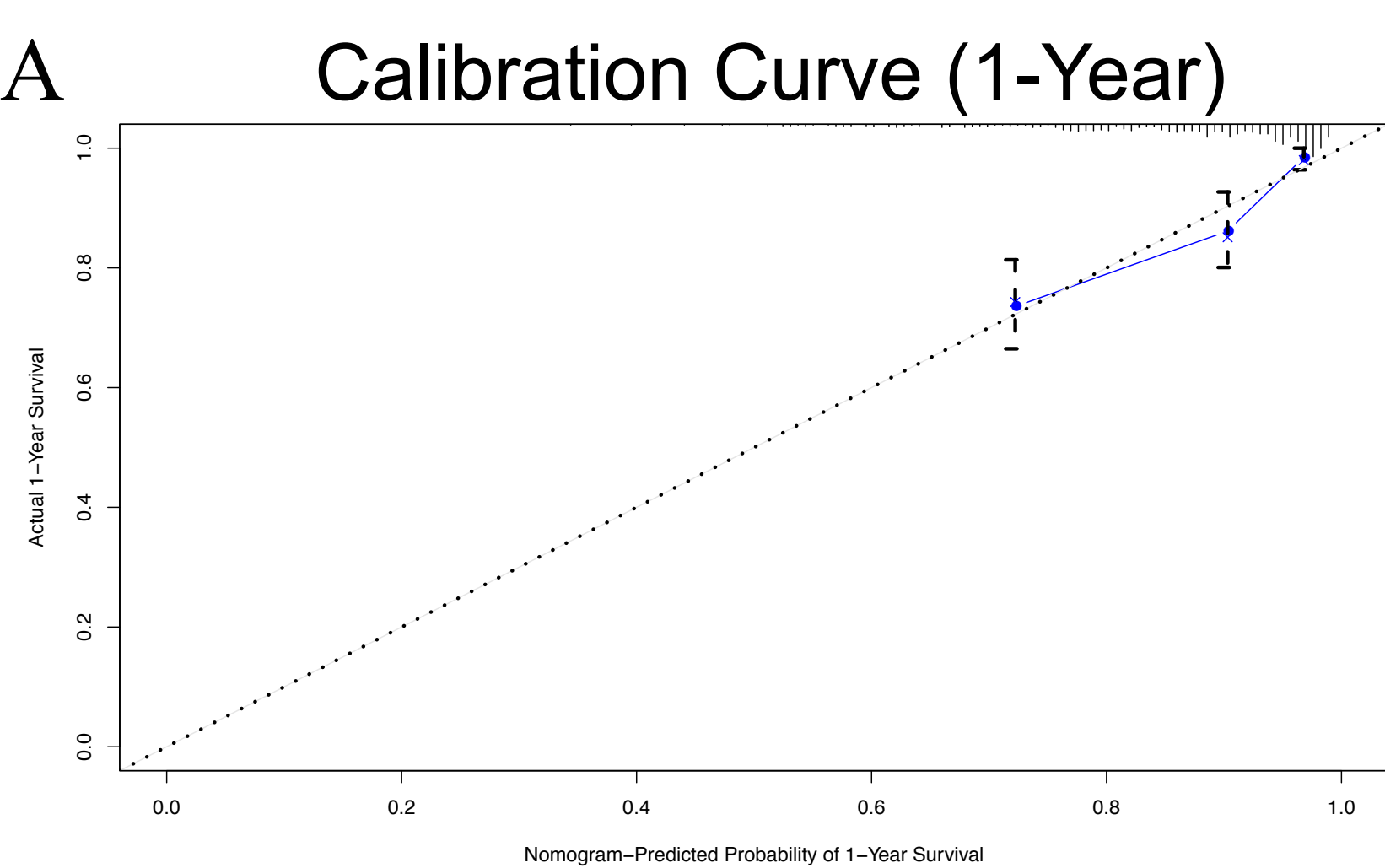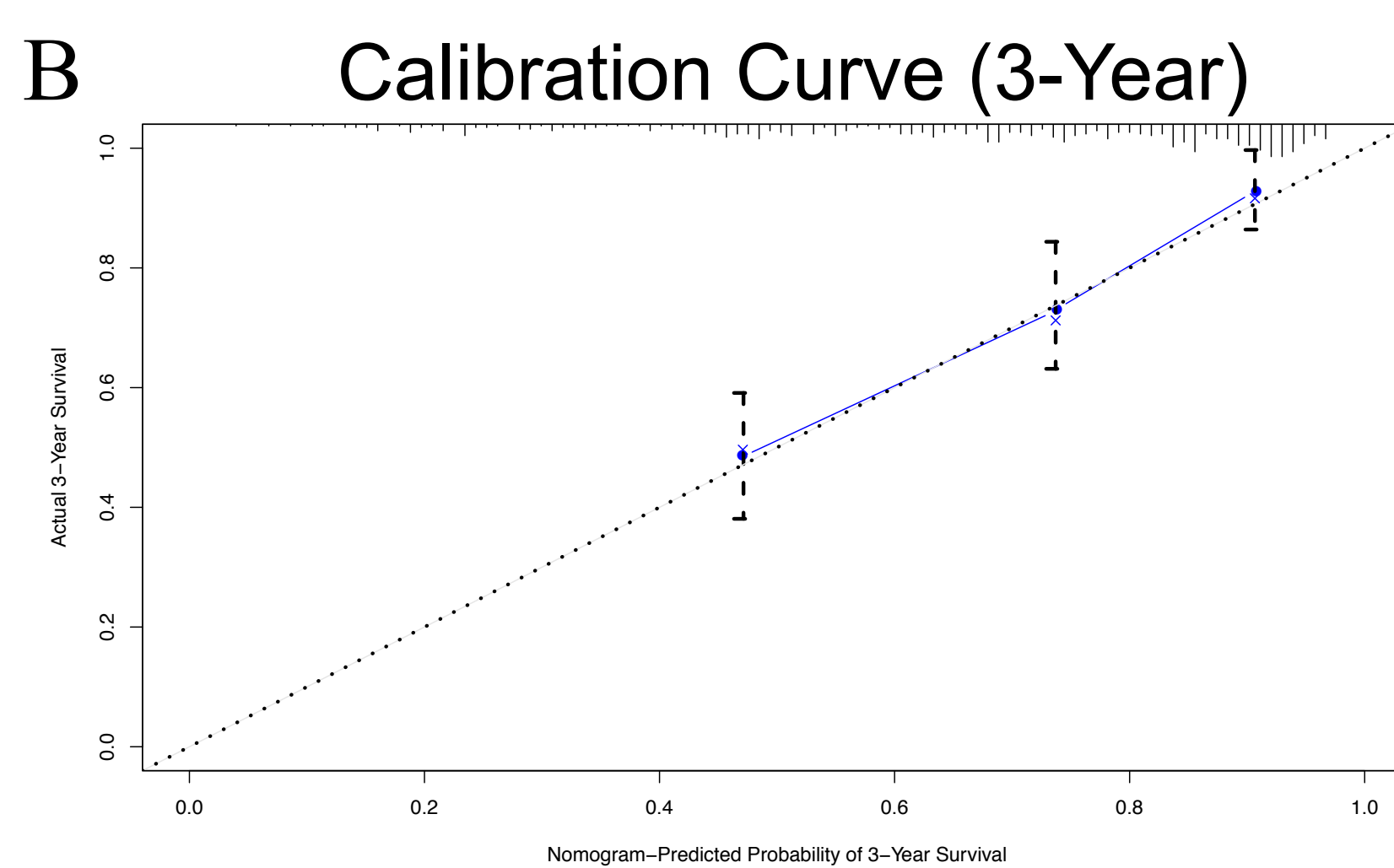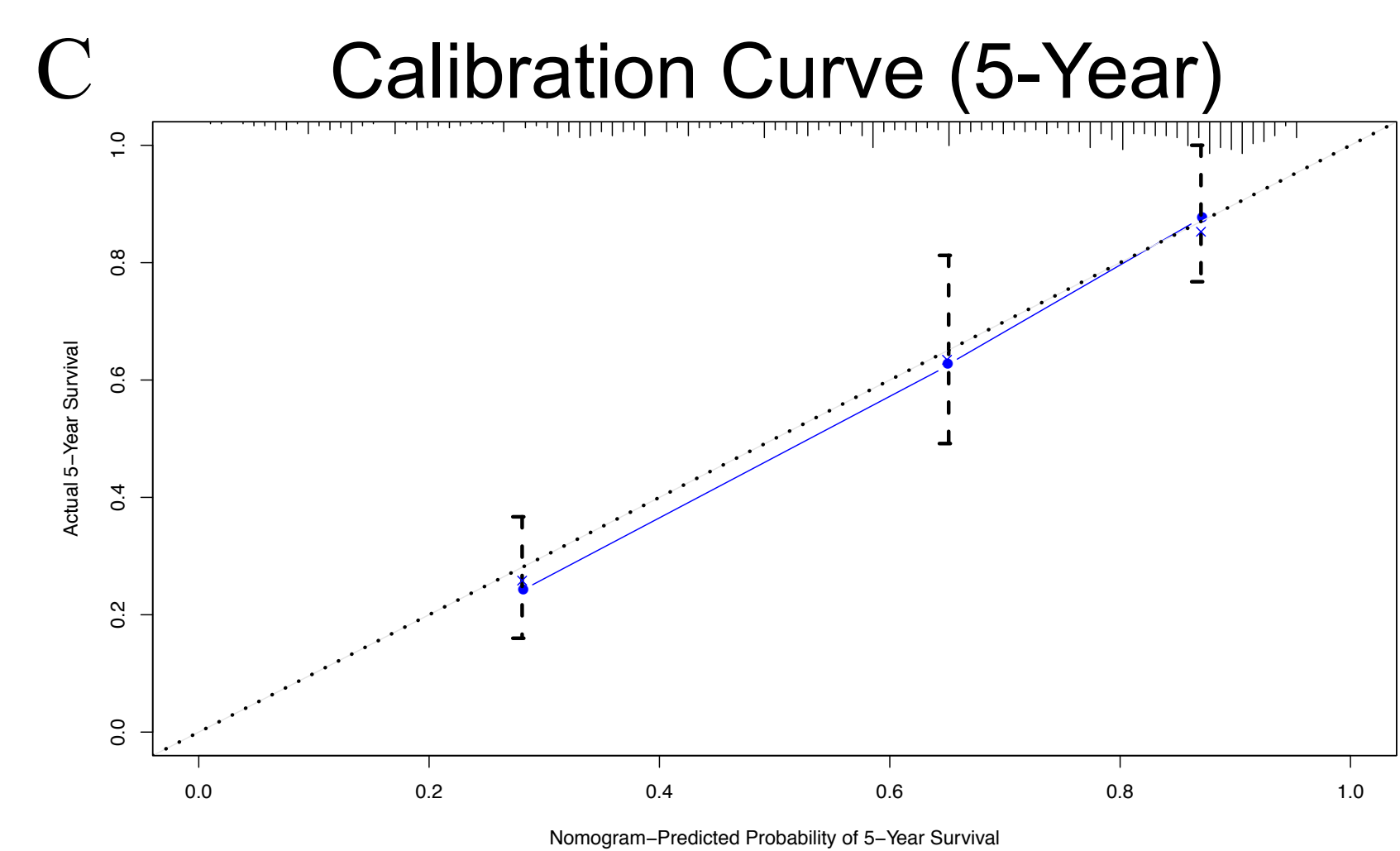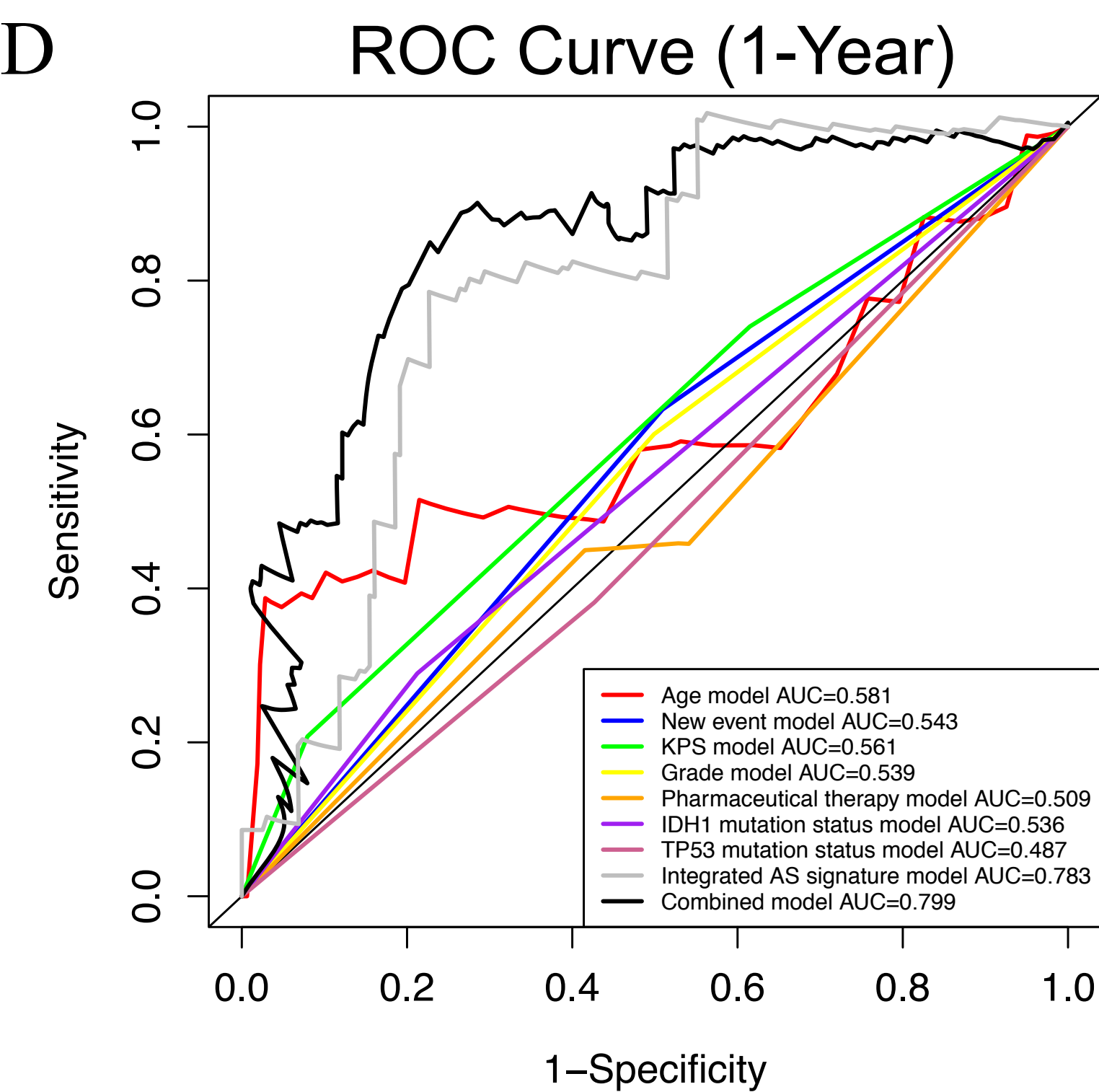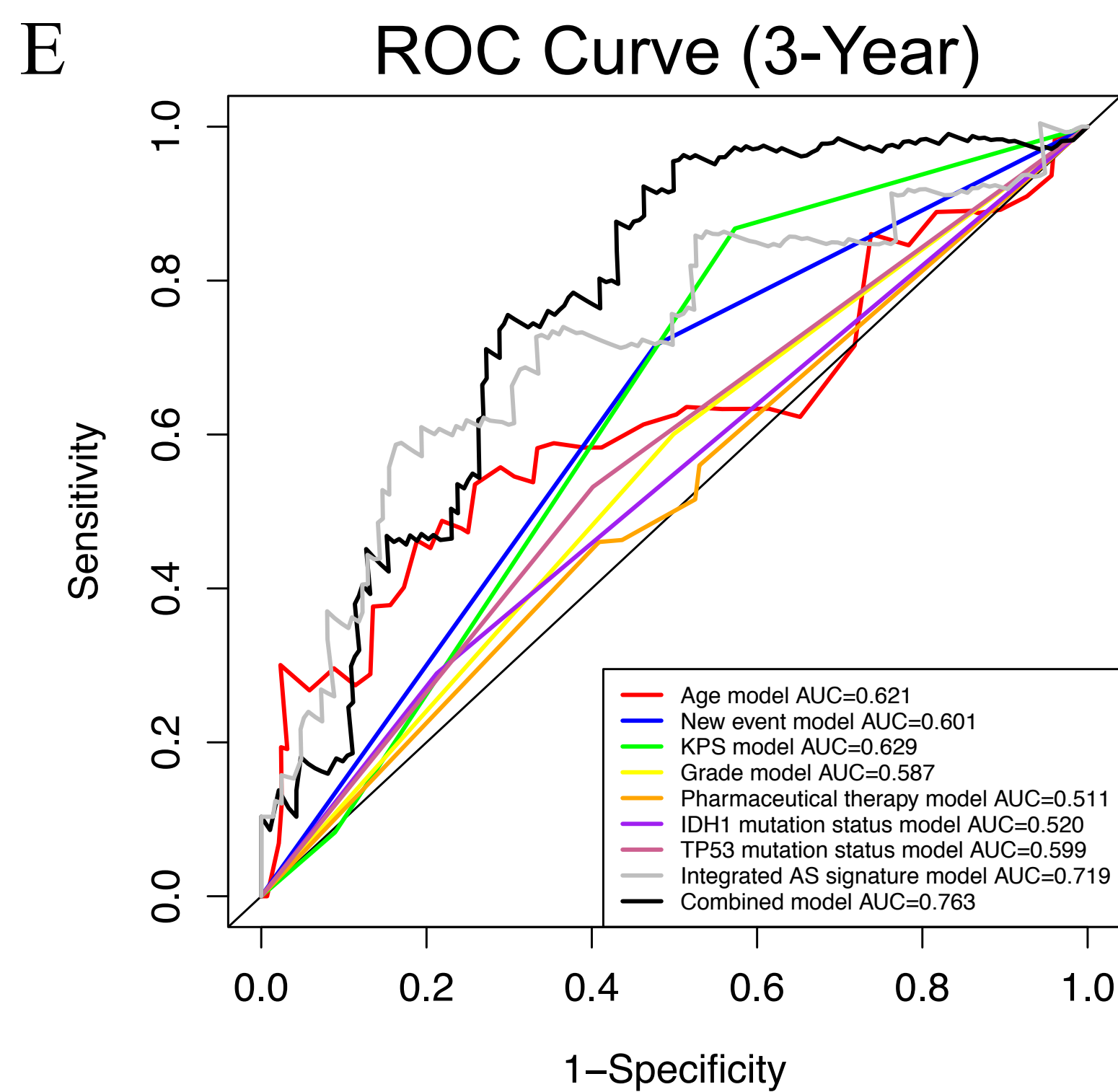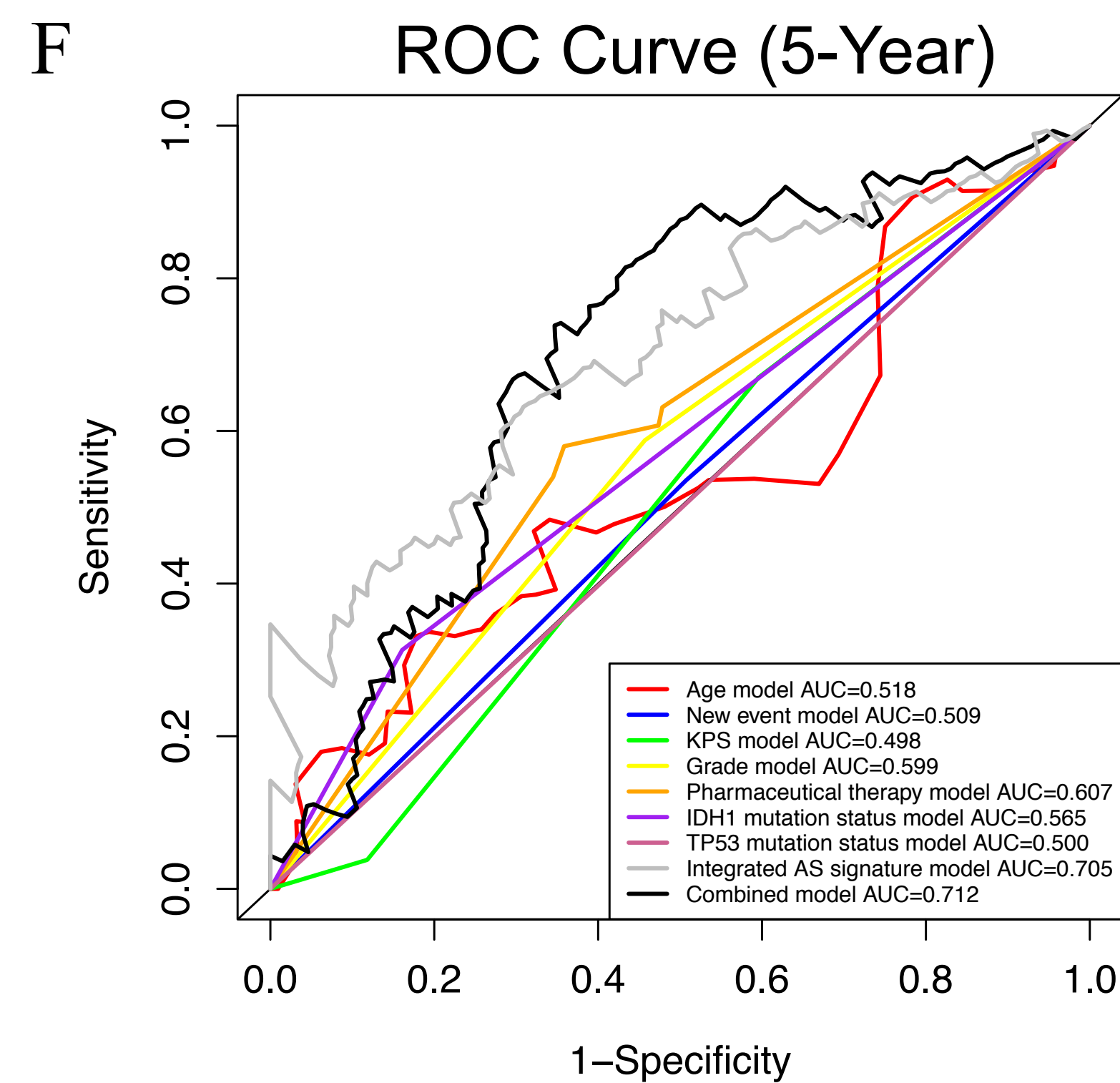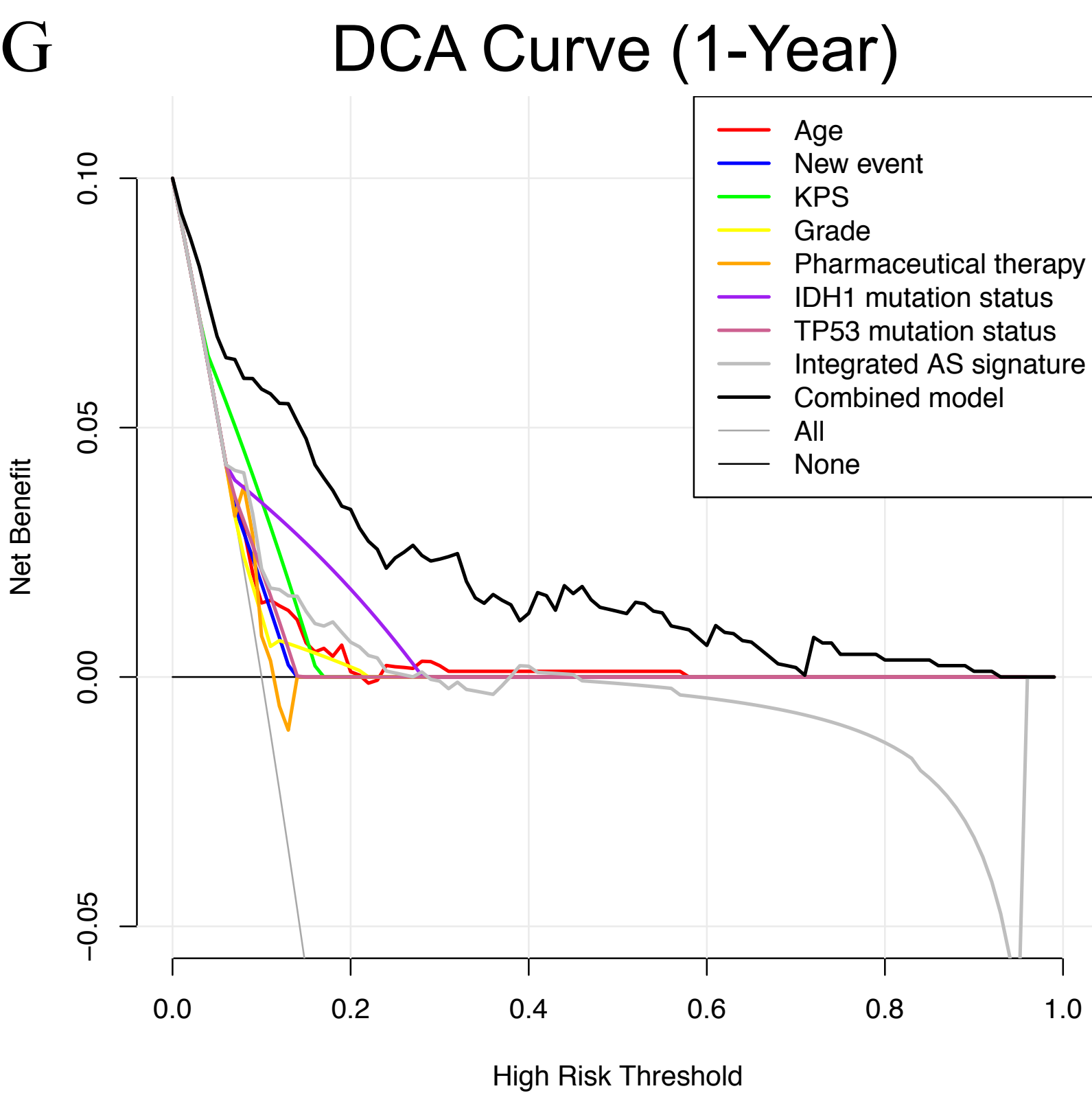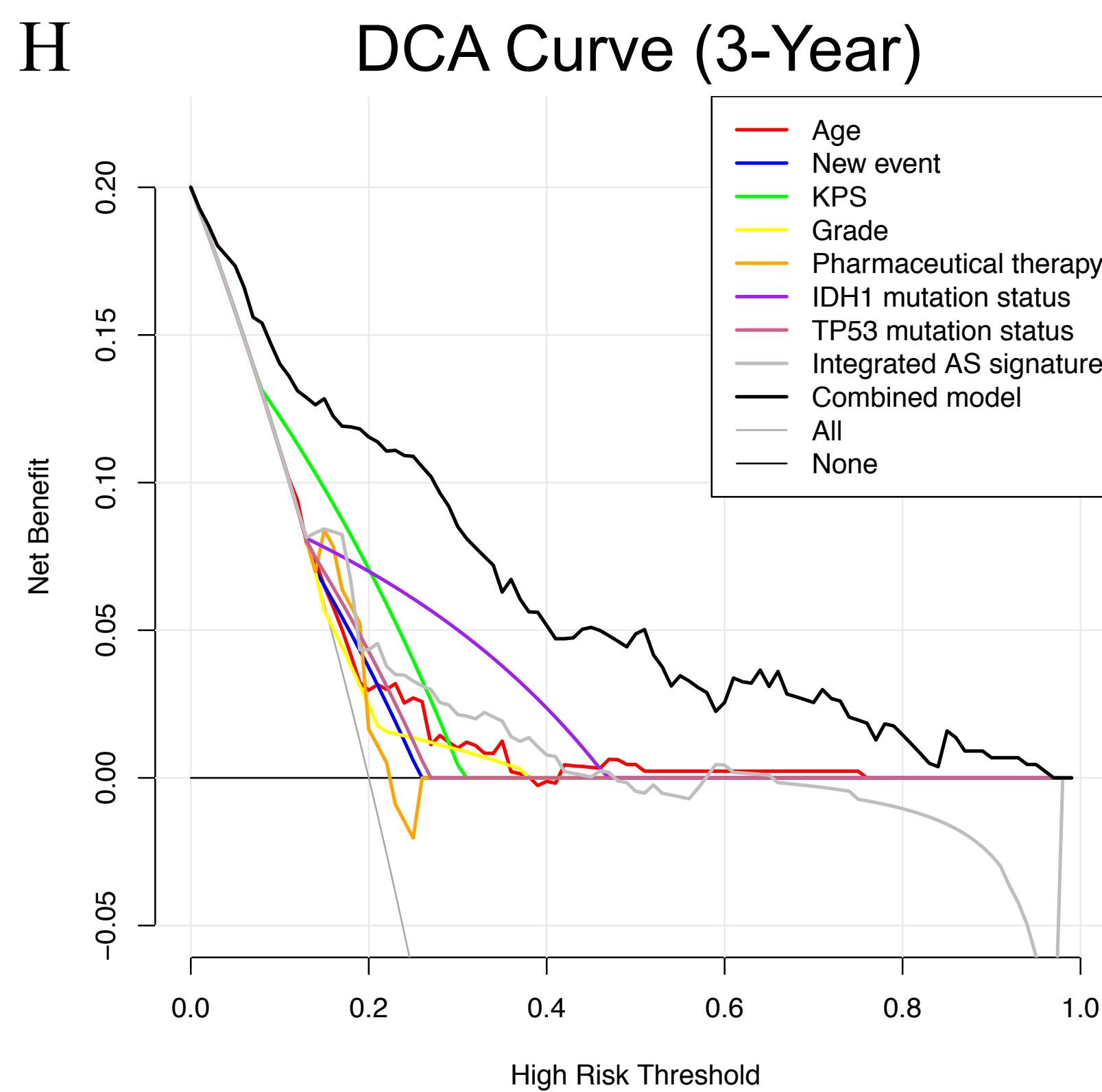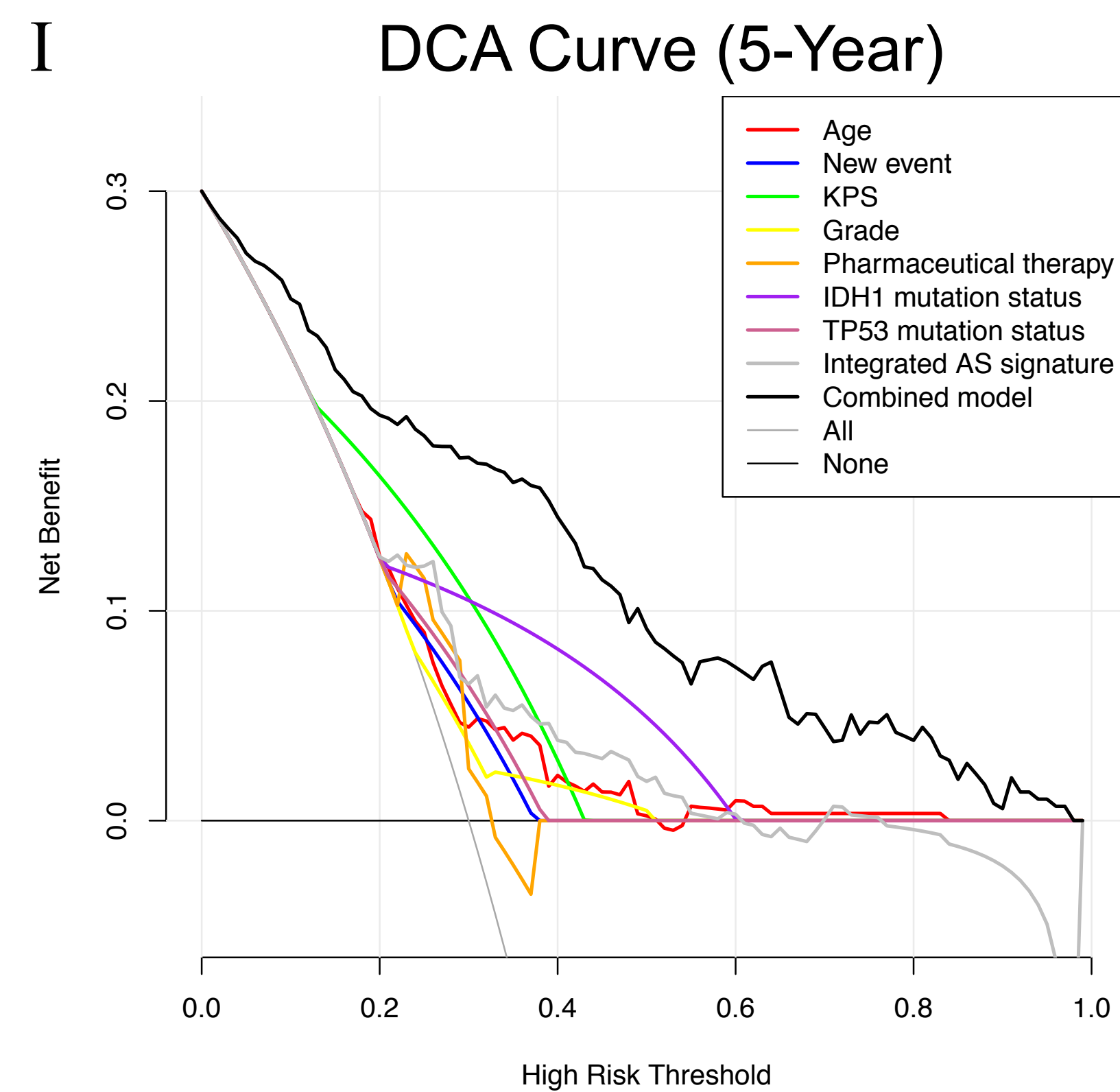

Supplement: Supplementary file 3 — Fig S3 [file CAM4-9-9266-s003.pdf]
