# Supplementary material for: Development of a nomogram for prognostic prediction of lower‐grade glioma based on alternative splicing signatures
Source: Cancer Med. 2020 Oct 13;9(24):9266–81. doi: 10.1002/cam4.3530 (PMC7774734; doi:10.1002/cam4.3530)
Supplement: Supplementary file 4 — Table S1 [file CAM4-9-9266-s004.docx]

Supplementary Table 1. Functional annotation and pathway enrichment analyses of the source genes of prognostic AS events.

| Category | Term | Description | Count | Gene Ratio | P Value |
| --- | --- | --- | --- | --- | --- |
| BP | GO:0006351 | transcription, DNA-templated | 178 | 0.075193052 | 0.012411305 |
| BP | GO:0006355 | regulation of transcription, DNA-templated | 140 | 0.059140603 | 0.014101609 |
| BP | GO:0043547 | positive regulation of GTPase activity | 76 | 0.032104899 | 2.64E-06 |
| BP | GO:0007155 | cell adhesion | 71 | 0.029992734 | 0.027184109 |
| BP | GO:0006915 | apoptotic process | 59 | 0.02492354 | 0.017289283 |
| CC | GO:0005634 | nucleus | 514 | 0.217130498 | 2.83E-10 |
| CC | GO:0005737 | cytoplasm | 485 | 0.204879945 | 3.40E-08 |
| CC | GO:0005829 | cytosol | 334 | 0.14109258 | 4.57E-09 |
| CC | GO:0005654 | nucleoplasm | 307 | 0.129686893 | 8.69E-13 |
| CC | GO:0016020 | membrane | 216 | 0.091245501 | 2.96E-05 |
| MF | GO:0005515 | protein binding | 838 | 0.35399875 | 2.57E-19 |
| MF | GO:0003677 | DNA binding | 150 | 0.063364931 | 0.044758684 |
| MF | GO:0005524 | ATP binding | 146 | 0.0616752 | 0.002793125 |
| MF | GO:0044822 | poly(A) RNA binding | 104 | 0.043933019 | 0.049992655 |
| MF | GO:0003676 | nucleic acid binding | 102 | 0.043088153 | 0.002487216 |
| KEGG | hsa01100 | Metabolic pathways | 107 | 0.045200318 | 0.089821743 |
| KEGG | hsa04810 | Regulation of actin cytoskeleton | 30 | 0.012672986 | 0.001440229 |
| KEGG | hsa04144 | Endocytosis | 28 | 0.011828121 | 0.030956053 |
| KEGG | hsa04015 | Rap1 signaling pathway | 25 | 0.010560822 | 0.032964272 |
| KEGG | hsa04510 | Focal adhesion | 24 | 0.010138389 | 0.045523837 |

AS, alternative splicing; BP, biological process; CC, cellular component; MF, molecular function; GO, Gene Ontology; KEGG, Kyoto Encyclopedia of Genes and Genomes pathway.
